# Phosphorylation of ULK1 by AMPK is essential for mouse embryonic stem cell self-renewal and pluripotency

**DOI:** 10.1038/s41419-017-0054-z

**Published:** 2018-01-18

**Authors:** Jiaqi Gong, Haifeng Gu, Lin Zhao, Liang Wang, Pinglei Liu, Fuping Wang, Haoyu Xu, Tongbiao Zhao

**Affiliations:** 1 State Key Laboratory of Stem Cell and Reproductive Biology, Institute of Zoology, Chinese Academy of Sciences, Beijing, 100101 China; 2 University of Chinese Academy of Sciences, Beijing, 100049 China; 3 Chinese Medicine Hospital in Linyi City, Linyi, 276600 China; 4 Hebei University, Baoding, 071002 China

## Abstract

Autophagy is a catabolic process to degrade both damaged organelles and aggregated proteins in somatic cells. We have recently identified that autophagy is an executor for mitochondrial homeostasis in embryonic stem cell (ESC), and thus contribute to stemness regulation. However, the regulatory and functional mechanisms of autophagy in ESC are still largely unknown. Here we have shown that activation of ULK1 by AMPK is essential for ESC self-renewal and pluripotency. Dysfunction of Ulk1 decreases the autophagic flux in ESC, leading to compromised self-renewal and pluripotency. These defects can be rescued by reacquisition of wild-type ULK1 and ULK1(S757A) mutant, but not ULK1(S317A, S555A and S777A) and kinase dead ULK1(K46I) mutant. These data indicate that phosphorylation of ULK1 by AMPK, but not mTOR, is essential for stemness regulation in ESC. The findings highlight a critical role for AMPK-dependent phosphorylation of ULK1 pathway to maintain ESC self-renewal and pluripotency.

Autophagy is a highly conserved lysosome-mediated degradation process in eukaryote cells^1–3^. The primary roles of autophagy in many organisms are first identified as an adaptive mechanism to nutrient deprivation. In yeast, autophagy-defective cells rapidly die upon starvation^4^. Mice deficient for Atg3, Atg5, and Atg7 have short survival time after birth upon food deprivation^5–7^. Recently, more and more studies have revealed that constitutive autophagy plays critical roles for cellular homeostasis and development. Dysfunction of autophagy leads to various diseases, such as neurodegeneration disease, hepatic failure, muscle atrophy, severe anaemia, and cancer^8,9^.

In contrast to the function study of autophagy in somatic cells, the role of autophagy in the regulation of pluripotent stem cell (PSC), including embryonic stem cell (ESC) and induced pluripotent stem cell (iPSC), is poorly understood. PSC is defined by the characters of self-renewal and pluripotency, which make it an unlimited resource for cell therapy and drug discovery^10–13^. Extensive studies have focused on mechanisms of transcription factors^14^, epigenetic factors^15^, and mircoRNAs^16^ in ESC stemness regulation; however, how the ESC maintain their self-renewal and pluripotency through metabolic regulation is largely unknown. Recently, we have identified the catabolic process autophagy as an executor to degrade the mitochondria in ESC, and thus maintain their mitochondrial homeostasis. Dysfunction of autophagy by Atg3 deletion inhibits mitochondrial removal in ESC, resulting in accumulation of abnormal mitochondria and attenuated pluripotency gene expression^17,18^. These data suggest that autophagy plays critical roles for ESC self-renewal and pluripotency.

Recent studies have identified that the kinases may play roles in ESC identity maintenance^19–21^. Serine/threonine protein kinase ULK1 is required for autophagy induction in yeast^22–25^. In mammalians, ULK1 formed complex with ATG13 and FIP200^26^. Both ATG13 and FIP200 are required for ULK1 localization to the isolation membrane in sensing autophagic signals^27^. Recently, Kim et al. reported that ULK1 was regulated via opposing phosphorylation by AMPK and mTOR. Under glucose depletion, AMPK promotes autophagy by directly activating ULK1 through phosphorylation of Ser(317), Ser(777)^28^, and Ser(555)^29^. In contrast, when nutrients are sufficient, mTOR prevents ULK1 activation by phosphorylating ULK1 at Ser(757), and thus disrupts the interaction between ULK1 and AMPK^28^. As a critical autophagy-initiating kinase, how ULK1 is regulated, and thus contribute to ESC stemness modulation, is unclear.

## Results

### Ulk1 deficiency inhibits ESCs self-renewal

**Fig. 1:**
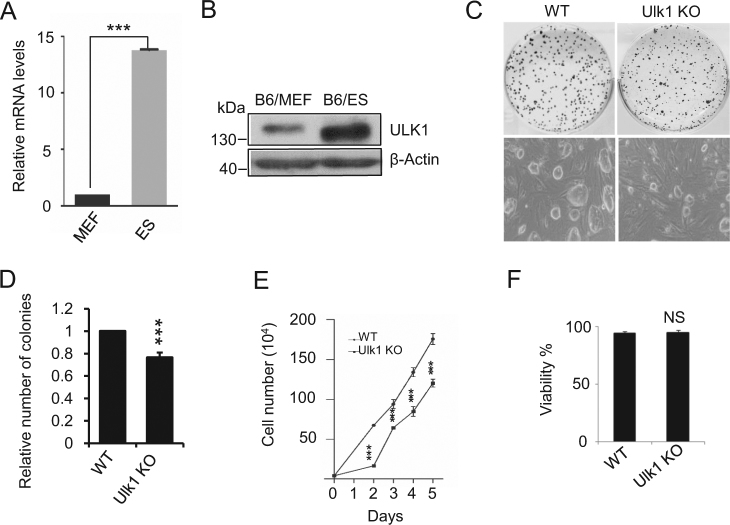
**ULK1 is essential for ESC self-renewal.** **a** The mRNA level of Ulk1 in ESC and MEF. Data shown as mean ± standard deviation (SD), *n* = 3; ***P < 0.001; Student’s *t* test. **b** Western blot analysis of whole cell extracts from MEF and ESC. β-actin served as a loading control. Images are representative of three independent experiments. **c**, **d** The colony formation assay of wild-type (WT) and Ulk1^−/−^ ESCs. Alkaline phosphatase (AP) staining and phase contrast images of ESC colonies. Data normalized to WT ESCs and shown as mean ± SD, *n* = 3; ***P < 0.001, Student’s *t* test. **e** The growth curves of WT and Ulk1^−/−^ ESCs. **f** Cells staining with PI and Annexin V, double negative as referred to viable cells and counted by a FACS. Data shown as mean ± SD, *n* = 3; NS not significant, Student’s *t* test

ULK1 is a member of serine/threonine kinase family. Quantitative PCR and western blot assays identified that Ulk1 is highly expressed in ESC at both mRNA and protein levels, compared to mouse embryonic fibroblast (MEF) (Fig. 1a, b). To examine whether ULK1 plays an important role in maintaining ESC stemness, we knocked-out the Ulk1 in vivo by using CRISPR-Cas9 system. The sgRNA-targeted sequence overlaps with the recognition sequence of the restriction enzyme Ehe I (Supplementary Fig. 1A). The restriction site will be destroyed by CRISPR-Cas9 if the targeting succeeds. We screened the Ulk1 knockout ES cell lines by Ehe I digestion first, and then the selected positive colonies were verified by DNA sequencing. Western blotting confirmed the silence of ULK1 protein expression in Ulk1 knockout ES lines (Supplementary Fig. 1B).

To test whether ULK1 plays a role for ESC self-renewal, we performed colony formation assays using both Ulk1^+/+^ and Ulk1^−/−^ ESCs. In contrast to the wild-type ESC, Ulk1^−/−^ ESC showed significantly decreased colony formation, indicating the compromised self-renewal ability (Fig. 1c, d). Correspondingly, the total number of Ulk1^−/−^ ESCs is significantly decreased compared to WT ESCs during culture (Fig. 1e). Cell cycle analysis identified that ULK1 deletion enlarged the G1/G2-M ratio (Supplementary Fig. 1I, J). However, ULK1 deletion did not enhance apoptosis (Fig. 1f). These results indicate that ULK1 is essential for ESC self-renewal.

In mammalians, ULK1 and ULK2 have been reported to be necessary for autophagy induction among the five ULK family members^30–32^. To investigate whether ULK2 has a similar function as ULK1 in ESC, we generated the Ulk2 knockout ES lines as well (Supplementary Fig. 1C). Surprisingly, although Ulk1 knockout dramatically decreased the autophagic flux, Ulk2 deletion did not affect autophagic flux any more in ESC (Supplementary Fig. 1D, E). Correspondingly, silencing Ulk2 did not affect ESC self-renewal and pluripotent gene expression (Supplementary Fig. 1F, G). These data indicate that Ulk2 is not involved in autophagy induction and thereafter pluripotency regulation in ESCs.

### Ulk1 deficiency impairs ESC pluripotency and differentiation

**Fig. 2:**
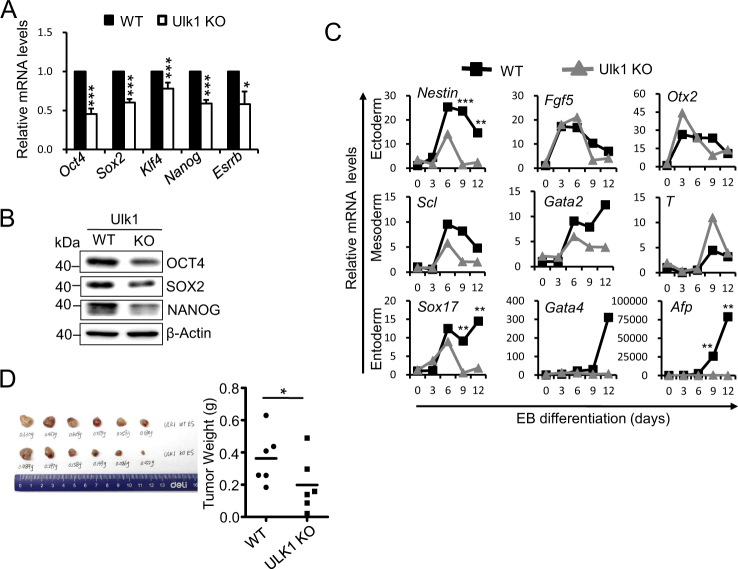
**ULK1 deficiency impairs ESC pluripotency and differentiation. a** ESC pluripotency was impaired by ULK1 deletion. The relative mRNA expression of pluripotent genes was detected by quantitative PCR between WT and Ulk1^−/−^ ESCs. Data normalized to WT ESCs and shown as mean ± SD, *n* = 3; ***P < 0.001, *P < 0.05, Student’s *t* test. **b** Western blotting for OCT4, SOX2, and NANOG between WT and Ulk1^−/−^ ESCs. β-actin served as a loading control. Images are representative of three independent experiments. **c** Lack of ULK1 impaired ESC lineage specification. The relative mRNA expression of genes representative of ectoderm, mesoderm, and endoderm were detected by quantitative PCR at different embryonic body (EB) differentiation days. Data from three independent experiments and shown as mean ± SD. *n*=3; **P < 0.01, ***P < 0.001, Student’s *t* test. **d** ULK1 deletion compromised ESC teratoma differentiation. Data from three independent experiments and shown as mean ± SD. *n*=3; *P < 0.05, Student’s *t* test

To test whether loss of ULK1 will affect the ESC pluripotency, we performed qPCR analysis on the mRNA expression of pluripotent genes in both Ulk1^+/+^ and Ulk1^−/−^ ESCs. From three independent experiments, we found that the expression of pluripotent genes in Ulk1^−/−^ ESCs was significantly decreased compared to wide-type ESC, suggesting that deletion of Ulk1 leads to the compromised pluripotency in ESC (Fig. 2a, b). In support of this assumption, Ulk1^−/−^ ESC showed abnormal embryonic body (EB) differentiation, characterized by delayed expression of certain endodermic and ectodermic marker genes (Fig. 2c). Then, we performed neuronal stem cell (NSC) differentiation in vitro and found lack of ULK1 dramatically inhibited NSC differentiation (Supplementary Fig. 2A, B).

Furthermore, teratoma formation assay was employed to investigate the Ulk1 contribution to ESC differentiation. While both Ulk1^+/+^ and Ulk1^−/−^ ESCs formed teratomas, the average weight of teratomas formed by Ulk1^−/−^ ESC is significantly lower than that of Ulk1^+/+^ ESC (Fig. 2d), supporting that Ulk1 is critical for differentiation of pluripotent stem cells. Together, these data suggest that ULK1 is essential for ESC pluripotency and differentiation.

### ULK1 regulates ESC self-renewal and pluripotency depending on its kinase activity

**Fig. 3:**
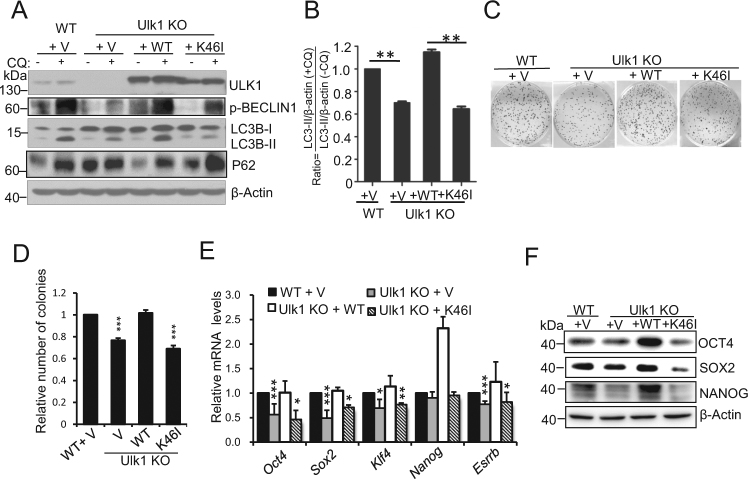
**ULK1 depends on the kinase activity to regulate ESC identity. a** Defective autophagy in Ulk1^−/−^ ESCs was rescued by reacquisition of WT, but not K46I mutant Ulk1 expression. Western blotting was performed in ULK1 rescue ESC lines using anti-ULK1 anti-P62, anti-p-BECLIN1, and anti-LC3 antibodies. β-actin served as the loading control. Samples were treated with or without chloroquine (100 μM) for 4 h. Images are representative of three independent experiments. **b** Quantification of the LC3 turnover ratio by calculating the ratio of LC3-II with or without CQ treatment in **a**. Data normalized to WT ESCs and shown as mean ± SD, *n*=3; **P < 0.01, Student’s *t* test. **c**, **d** Reacquisition of wild-type, but not K46I mutant Ulk1 expression in Ulk1^−/−^ cells compensated for the self-renewal. Data normalized to WT ESCs and shown as mean ± SD, *n* = 3; ***P < 0.001; Student’s *t* test. **e** Reacquisition of WT, but not K46I mutant Ulk1 expression in Ulk1^−/−^ cells compensated for the pluripotency. The relative mRNA expression of pluripotent genes was detected by quantitative PCR in WT and Ulk1 rescue ESC lines. Data normalized to WT ESC harboring empty vector and shown as mean ± SD, *n* = 3; *P < 0.05; **P < 0.01; ***P < 0.001; Student’s *t* test. **f** Defective pluripotency gene expression in Ulk1^−/−^ ESCs was rescued by reacquisition of WT, but not K46I mutant Ulk1 expression. Western blotting was performed in ULK1 rescue ESC lines using anti-OCT4, anti-SOX2, and anti-NANOG antibodies. β-actin served as the loading control. Images are representative of three independent experiments

Previous study reported that K46I mutant of ULK1 markedly inhibited its kinase activity and failed to form the autophagosomes in starved 293/GFP-LC3 cells^33^. To investigate whether the aberrant self-renewal and pluripotency of Ulk1^−/−^ ESC were directly caused by the loss of ULK1 kinase activity, gain-of function assays were performed by introducing Ulk1 expression into Ulk1^−/−^ ESC. We established stable Ulk1^−/−^ ESC lines carrying an empty vector, wild-type Ulk1 or kinase dead mutant Ulk1 (K46I). As expected, autophagic flux can only be rescued in Ulk1^−/−^ stable ESC lines carrying wild-type Ulk1, but not the empty vector or Ulk1 (K46I) mutant, indicating kinase activity is important for Ulk1 to induce autophagy in ESC (Fig. 3a, b). Correspondingly, defective colony formation ability in Ulk1^−/−^ ESC was only restored by gain of wild-type Ulk1, but not Ulk1 (K46I) mutant (Fig. 3c, d). Meanwhile, the decreased pluripotency gene expression in Ulk1^−/−^ ESCs was only rescued by reacquisition expression of wide-type, but not K46I mutant, Ulk1 (Fig. 3e, f). Together, these data suggest that ULK1 depends on its kinase activity to regulate the ESC self-renewal and pluripotency.

### Phosphorylation of ULK1 by AMPK, but not mTOR, is required for ESC self-renewal and pluripotency

**Fig. 4:**
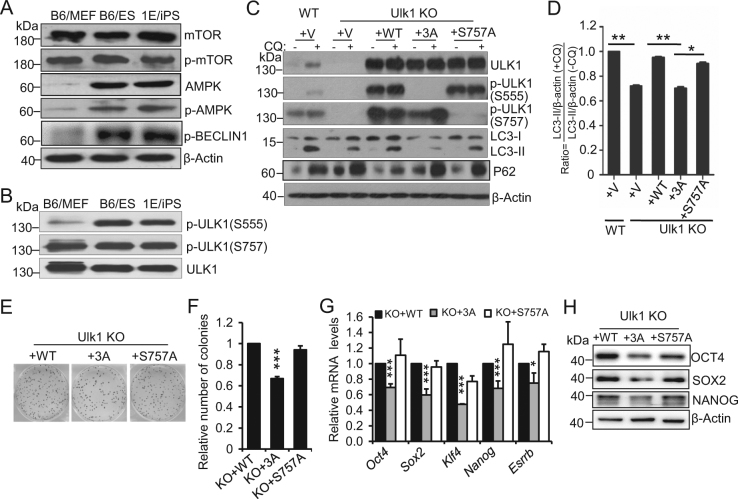
**Phosphorylation of ULK1 by AMPK, but not mTOR is required for ESC self-renewal and pluripotency. a** AMPK, phosphorylated AMPK (p-AMPK) and phosphorylated BECLIN1 (p-BECLIN1), is highly expressed in ESCs than in MEFs. Whole cell extracts from MEFs, ESCs, and iPSCs were harvested for western blot using anti-mTOR, anti-p-mTOR, anti-AMPK, anti-p-AMPK, anti-p-BECLIN1, and anti-β-actin antibodies. **b** Phosphorylation of ULK1 at Ser555, but not at Ser757, is higher in ESCs than MEFs. Whole cell extracts from MEFs, ESCs, and iPSCs were harvested for western blot using anti-p-ULK1(S555), anti-p-ULK1(S757), and anti-ULK1 antibodies. ULK1 serves as a loading control. **c** Defective autophagy in Ulk1^−/−^ ESCs was rescued by reacquisition of WT and S757A mutant, but not 3A-mutant Ulk1 expression. Western blotting was performed in ULK1 rescue ESC lines using anti-ULK1, anti-p-ULK1(S555), anti-p-ULK1(S757), anti-LC3, and anti-P62 antibodies. β-actin served as the loading control. The samples were treated with or without chloroquine (100 μM) for 4 h. **d** Quantification of the LC3 turnover ratio by calculating the ratio of LC3-II with or without CQ treatment in **c**. Data normalized to WT ESCs and shown as mean ± SD, *n* = 3; **P < 0.01, *P < 0.05, Student’s *t* test. **e**, **f** Reacquisition of wild-type and S757A mutant, but not 3A-mutant Ulk1 expression in Ulk1^−/−^ cells compensated for the self-renewal. Data shown as mean ± SD, *n* = 3; ***P < 0.001; Student’s *t* test. **g** Reacquisition of wild-type and S757A mutant, but not 3A-mutant Ulk1 expression in Ulk1^−/−^ cells compensated for the pluripotency. Data shown as mean ± SD, *n* = 3; *P < 0.05; **P < 0.01; ***P < 0.001; Student’s *t* test. **h** Defective pluripotency gene expression in Ulk1^−/−^ ESCs was rescued by reacquisition of WT and S757A mutant, but not 3A-mutant Ulk1 expression. Western blotting was performed in ULK1 rescue ESC lines using anti-OCT4 anti-SOX2 and anti-NANOG antibodies. β-actin served as the loading control. Images are representative of three independent experiments. Images in **a**, **b**, **c**, and **h** are representative of three independent experiments

Ulk1 can be coordinately regulated by AMPK and mTOR through their kinase activity in somatic cells. To understand how the ULK1 was regulated in ESC, and thus contributed to stemness regulation, we first detected the activation status of mammalian target of rapamycin (mTOR), the principal pathway restricting autophagy induction, and adenosine monophosphate-activated protein kinase (AMPK), a recently identified autophagy activator, in both ESC and MEF. We did not observe differences in mTOR activation level between MEF and pluripotent stem cells using western blot detection for phosphorylated mTOR (p-mTOR). In contrast, the activation of AMPK (p-AMPK), ATG14 (p-ATG14), and BECLIN1 (p-BECLIN1) in pluripotent stem cells are significantly higher than that of MEF (Fig. 4a and Supplementary Fig. 1H), indicating AMPK activation is essential for stemness regulation in ESC. We then investigated the activation of ULK1 by either AMPK or mTOR between somatic MEF and pluripotent stem cells, respectively. Interestingly, ULK1 phosphorylation on Ser(555) by AMPK, but not on Ser(757) by mTOR, was detected to be significantly higher in pluripotent stem cells than in somatic MEF. These data indicate that phosphorylation of ULK1 by AMPK, but not mTOR, is essential for stemness regulation in ESC (Fig. 4b).

To investigate whether non-canonical function of ULK1 contributes to pluripotency regulation, we treated the cells with TrkA inhibitors. Inhibition of ULK1 target TrkA did not change pluripotent gene expression in ESCs, indicating that non-canonical function of ULK1 is not involved in ESC identity regulation (Supplementary Fig. 2C).

To further confirm that AMPK phosphorylation of ULK1 is crucial for maintaining the ESC self-renewal and pluripotency, we performed gain-of-function assays by establishing stable Ulk1^−/−^ ESC lines carrying an empty vector, wild-type Ulk1, 3A-mutant Ulk1 (S317A, S777A, and S555A), or S757A mutant Ulk1. Interestingly, decreased autophagic flux in Ulk1^−/−^ ESC can only be restored by reacquisition of wild-type and S757A mutant Ulk1, but not the empty vector or 3A-mutant Ulk1 (Fig. 4c, d). Correspondingly, reacquisition of wild-type and S757A mutant Ulk1, but not 3A-mutant Ulk1, in Ulk1^−/−^ ESC can restore the abnormal colony formation and pluripotency gene expression (Fig. 4e–h). Together, these data suggested that phosphorylation of ULK1 by AMPK, but not mTOR, is essential for ESC self-renewal and pluripotency.

## Discussion

Recently, we have demonstrated that ATG3-dependent autophagy plays critical roles in maintaining the ESC stemness. ATG1 is the first identified autophagy machinery gene in *Saccharomyces cerevisiae*. In mammals, five ATG1 homologous have been identified as ULK1, ULK2, ULK3, ULK4, and STK36. ULK1 and ULK2 are the most closely related members among the family^34^. ULK4 has been suggested to maintain neural stem cell pools in mice^35^. ULK1, *Saccharomyces cerevisiae* ATG1 kinase homolog in mammals, is an autophagy-initiating kinase, which can form a complex with ATG13, FIP200, and ATG101 to initiate autophagy upon nutrient deprivation in somatic cells. How the ULK1 is regulated in ESC, and thus contributes to stemness, is unknown. In this study, we found that ULK1 is highly expressed in ESC, and its kinase activity is critical for stemness regulation. We further demonstrated that phosphorylation of ULK1 by AMPK, but not mTOR, is required for ESC self-renewal and pluripotency.

In somatic cells, mTOR and AMPK coordinate to regulate cellular nutrition and energy signals to maintain cellular homeostasis under different nutrient conditions^36,37^. Under nutrient-rich conditions, activated mTOR phosphorylates ULK1 on Ser(757) and disrupt ULK1–AMPK interaction, maintaining ULK1 inactivation^28^. When cellular energy is decreasing like glucose deprivation, AMPK is activated and phosphorylates ULK1 at Ser(555), leading to disrupting the interaction between mTOR and ULK1^38,39,28^. In contrast, we found here that the phosphorylation level of mTOR is similar between somatic MEF and ESC, while the level of both AMPK and phosphorylated AMPK are significantly increased in ESC compared to somatic MEF under normal conditions. Most importantly, active phosphorylation of ULK1 by AMPK on Ser(555) is significantly higher in ESC than in somatic MEF. These data suggest that constitutive activation of ULK1 by AMPK is an intrinsic signal pathway inside ESC to regulate their identity under normal physiological conditions.

ESCs have a short cell cycle and maintain a rapid proliferation rate with a shortened G1 and an extended S phase^40,41^. In favor of the high proliferation rate, ESCs are expected to exert substantial energy demands and efficient material turnover. However, ESCs rely mainly on glycolysis and have less ATP generation than somatic cells^42^. The increased expression of both ULK1 and AMPK-phosphorylated ULK1 in ESCs compared to somatic fibroblasts suggests a high autophagic potency in ESCs. We propose that ESCs harness enhanced autophagic potency through elevating AMPK phosphorylation of ULK1 to balance energetic with biosynthetic demands to facilitate rapid cell duplication and self-renewal.

## Materials and methods

### Construction of Ulk1 expression vector

Mouse cDNA of Ulk1 was cloned into the pCDH-CAG-mCherry lentivirus-vector. The Ulk1 mutants 3A (S317A, S555A, and S777A), S757A, and K46I vectors were constructed by using overlap PCR. The primers we used were listed as follows: Ulk1-F, 5′-GGAATTCGCCACCATGGAGCCGGGCCGCGGC-3′; Ulk1-R, 5′-GCGCGGCCGCTCAGGCATAGACACCACTCA-3′; S317A-F, 5′-CTGGCCTCTCCACCGGCCCTGGGGGAGATGCCA-3′; S317AR, 5′-TGGCATCTCCCCCAGGGCCGGTGGAGAGGCCAG-3′; S555AF, 5′-GGCTGCCGCCTGCACGCTGCCCCTAACCTGTCC-3′; S555AR, 5′-GGACAGGTTAGGGGCAGCGTGCAGGCGGCAGCC-3′; S777AF, 5′-TTCTCAGTGGGCTCTGCCAGCTCCCTGGGCTCT-3′; S777AR, 5′-AGAGCCCAGGGAGCTGGCAGAGCCCACTGAGAA-3′; S757AF, 5′-GTATTTACTGTAGGCGCCCCACCCAGTGGTGCC-3′; S757AR, 5′-GGCACCACTGGGTGGGGCGCCTACAGTAAATAC-3′; K46IF, 5′-CTGGAGGTGGCCGTCATATGCATTAACAAGAAG-3′; and K46IR, 5′-CTTCTTGTTAATGCATATGACGGCCACCTCCAG-3′.

### Cell culture

The ESCs were isolated at embryonic d 3.5, which have been characterized previously^17^. ESCs were routinely maintained in ESC medium (knockout Dulbecco’s Modified Eagle’s Medium with 15% fetal bovine serum, 2 mM glutamine, 1 mM sodium pyruvate, 0.1 mM nonessential amino acids, 100 μg/ml streptomycin, 100 U/ml penicillin, 0.055 mM β-mercaptoethanol, and 1000 U/ml leukemia inhibitory factor). For colony formation assay, ESCs were seeded at 1000 cells/well in a 6-well plate and cultured as described previously^18^.

### Ulk1 knockout ESC

We designed and synthesized mouse Ulk1 sgRNA, and the sequence of Ulk1 sgRNA were as follows: F: ACCGGATTGGACACGGCGCCTTCG; R: AAACCGAAGGCGCCGTGTCCAATCC. After annealing, the oligos were ligated into the linearized vector px330, then we transfected ESC using this plasmid by electroporation. About 72 h after electroporation, we reseeded the cell on a 6-well plate, and then picking single colonies for genotyping. A region around the cutting site was amplified using flowing primers: F, 5′-CACCGGATTGGACACGGCGCCTTCG-3′; R, 5′-AAACCGAAGGCGCCGTGTCCAATCC-3′. PCR products were digested by *Ehe* I enzyme in order to identify the Ulk1 knockout ESCs.

### Lentivirus packing and stable ES cell line establishment

Lentiviral particles were produced by calcium phosphate-mediated co-transfection of HEK293T cells with psPAX2 and pMD2.G plasmids. After 48 h of transfection, we collected the virus supernatant and then infected ESC, which was seeded at 40,000 cells/well in a 6-well plate. After 72 h of infection, stable ES cell lines were selected by cell sorting for mCherry expression (BD Aria).

### Colony formation assay and AP staining

ESCs were seeded at 1000 cells/well in a 6-well plate, and cultured for about 7 days. The colonies were stained by an Alkaline Phosphatase Assay Kit (Beyotime), and the colony numbers were analyzed by an Image-Pro Plus software.

### Western blotting

ESCs were either treated or not treated with 100 μM Chloroquine (Sigma) for 4 h. After the treatments, the cells were collected and lysed by RIPA buffer with protease inhibitor PMSF (Beyotime). The lysates were resolved by SDS/PAGE, and proteins were transferred onto PVDF membranes. For WB, the following antibodies were used: the anti-actin (1:5000) was purchased from Abcam, anti-LC3B (1:2000) and anti-ULK1 (1:1000) were obtained from Sigma-Aldrich, anti-pULK1 (S555) (1:1000), and anti-pULK1 (S757) (1:1000) were purchased from Sigma, and anti-mTOR (1:1000), anti-mTOR (1:1000), anti-AMPK (1:1000), anti-p-AMPK (1:1000) were purchased from Cell Signaling Technology.

### Quantitative real-time PCR

Total RNA was extracted from ESCs and EBs with a total RNA isolation kit (GeneMark). Total RNA (2 μg) was reverse transcribed into cDNA using a SuperScript™ III first-strand synthesis system (Invitrogen). The primers used for testing the pluripotency and three germ layer gene expression were as follows: Oct4F, 5′-AGAGGATCACCTTGGGGTACA-3′; Oct4R, 5′-CGAAGCGACAGATGGTGGTC-3′; Sox2F, 5′-GCGGAGTGGAAACTTTTGTCC-3′; Sox2R, 5′-CGGGAAGCGTGTACTTATCCTT-3′; Klf4F, 5′-GTGCCCCGACTAACCGTTG-3′; Klf4R, 5′-GTCGTTGAACTCCTCGGTCT-3′; NanogF, 5′-TCTTCCTGGTCCCCACAGTTT-3′; NanogR, 5′-GCAAGAATAGTTCTCGGGATGAA-3′; EsrrbF, 5′-CAGGCAAGGATGACAGACG-3′; EsrrbR, 5′-GAGACAGCACGAAGGACTGC-3′; NestinF, 5′-CCCTGAAGTCGAGGAGCTG-3′; NestinR, 5′-CTGCTGCACCTCTAAGCGA-3′; Fgf5F, 5′-CTGTATGGACCCACAGGGAGTAAC-3′; Fgf5R, 5′-ATTAAGCTCCTGGGTCGCAAG-3′; Otx2F, 5′-TATCTAAAGCAACCGCCTTACG-3′; Otx2R, 5′-AAGTCCATACCCGAAGTGGTC-3′; SclF, 5′-CTGGCCTCCAGCTACATTTCT-3′; SclR, 5′-GTCACGGTCTTTGCTCAACTT-3′; GATA2F, 5′-CACCCCGCCGTATTGAATG-3′; GATA2R, 5′-CCTGCGAGTCGAGATGGTTG-3′; TF, 5′-GCTTCAAGGAGCTAACTAACGAG-3′; TR, 5’-CCAGCAAGAAAGAGTACATGGC-3′;AFPF, 5′-CTTCCCTCATCCTCCTGCTAC-3′; AFPR, 5′-ACAAACTGGGTAAAGGTGATGG-3′; GATA4F, 5′-CCCTACCCAGCCTACATGG-3′; GATA4R, 5′-ACATATCGAGATTGGGGTGTCT-3′; Sox17F, 5′-GATGCGGGATACGCCAGTG-3′; Sox17R, 5′-CCACCACCTCGCCTTTCAC-3′; ActinF, 5′-GGCTGTATTCCCCTCCATCG-3′; ActinR, and 5′-CCAGTTGGTAACAATGCCATGT-3′.

## Electronic supplementary material

Supplement figure
