# Supplementary material for: Phosphorylation of ULK1 by AMPK is essential for mouse embryonic stem cell self-renewal and pluripotency
Source: Cell Death Dis. 2018 Jan 18;9(2):38. doi: 10.1038/s41419-017-0054-z (PMC5833692; doi:10.1038/s41419-017-0054-z)
Supplement: Supplementary file 1 — Supplement figure [file 41419_2017_54_MOESM1_ESM.pdf]

Supplementary Figure 1. Ulk1 but not Ulk2 silence impaired ESC self-renewal and pluripotency

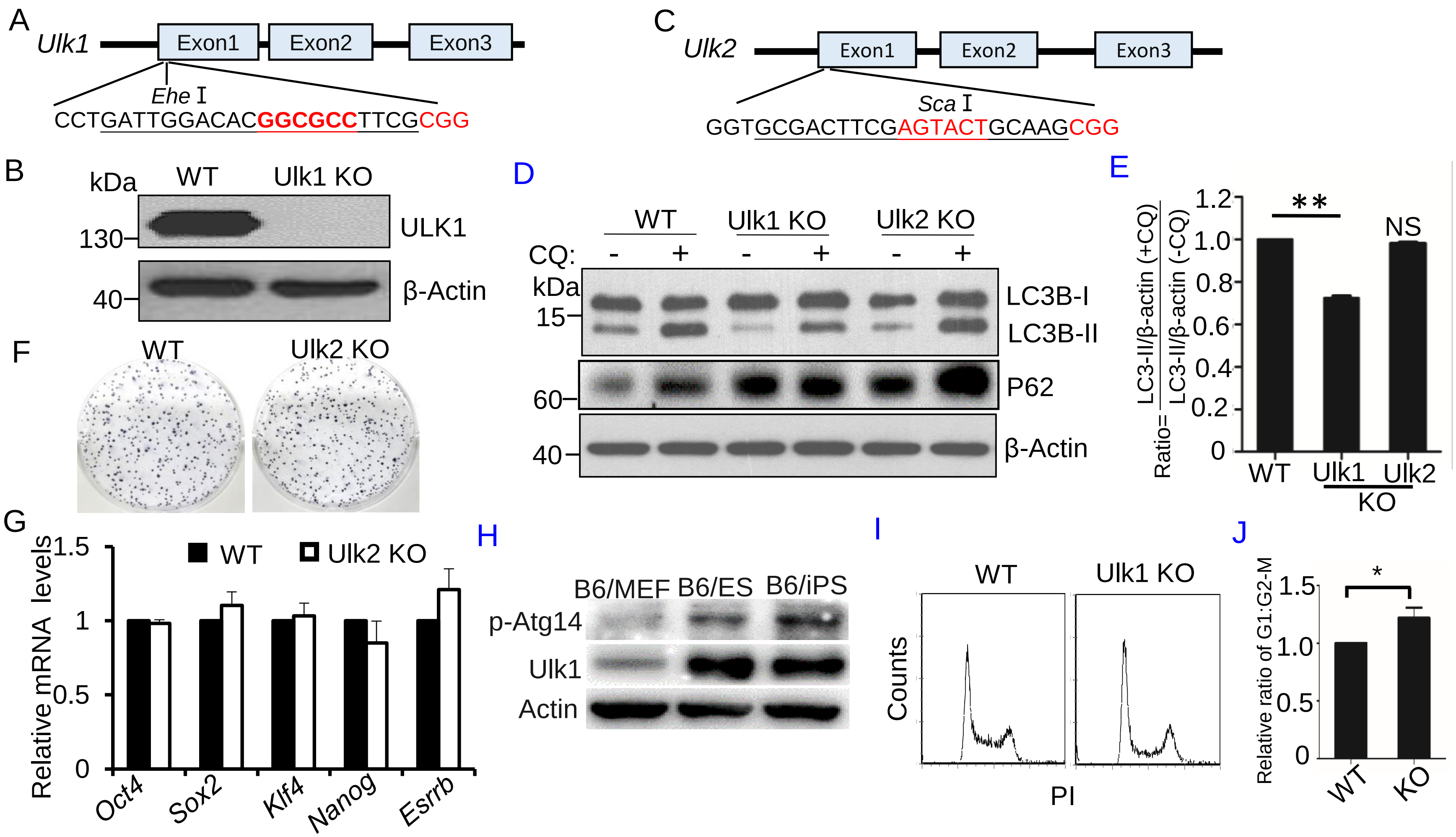

Supplement Figure 1. Ulk2 silence did not affect ESC self-renewal and pluripotency. **(A)** sgRNA designed for targeting the first exon of *Ulk1*. **(B)** Western blot analysis of whole cell extracts from *Ulk1*$^{+/+}$ and *Ulk1*$^{-/-}$, β-Actin served as a loading control. **(C)** sgRNA designed for targeting the first exon of Ulk2. **(D)** The autophagic flux was impaired in *Ulk1*$^{-/-}$ but not in *Ulk2*$^{-/-}$. Western blotting was performed in Ulk1$^{+/+}$, Ulk1$^{-/-}$ and Ulk2$^{-/-}$ ESC lines using anti-LC3 and anti-P62 antibodies. Samples were treated with or without Chloroquine (100 μM) for 4 h. β-Actin served as the loading control. **(E)** Quantification of the LC3 turnover ratio by calculating the ratio of LC3-II with or without CQ treatment in **(D)**. Data normalized to WT ESCs and shown as mean ± SD, n=3; **, P<0.01, Student's t-test. **(F)** Ulk2 deletion did not affect ESC colony formation ability. **(G)** Ulk2 deletion did not affect expression of pluripotent genes. **(H)** The phosphorylated Atg14 (p-Atg14) is highly expressed in ESCs than in MEFs. Whole cell extracts from MEFs, ESCs and iPSCs were harvested for western blot using anti-p-Atg14, anti-Ulk1 and anti-β-Actin antibodies. **(I)** PI staining of DNA contents of both WT and ULK1 KO ESCs**. (J)** ULK1 deletion enhanced G1/G2-M ratio.

Supplementary Figure 2. Ulk1 silence impaired the NSC differentiation from ESC

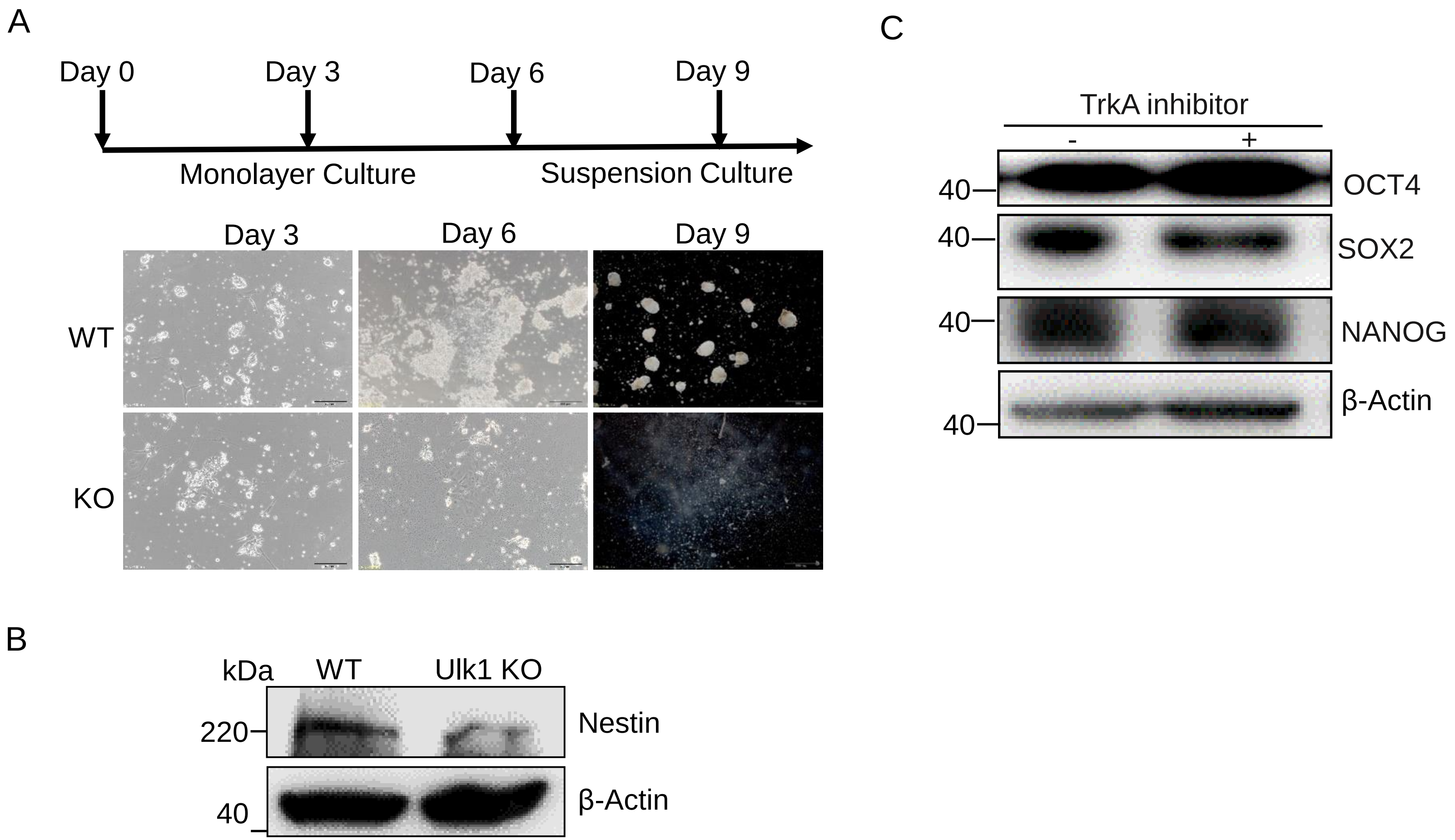

Supplement Figure 2. Ulk1 silence impaired the neural stem cell differentiation of mESCs in vitro. **(A)** Differentiation of mESCs into neural stem cell. Images were captured on days 3, 6 and day 9. (**B)** Western blotting for the Nestin between WT and Ulk1 KO ESC-derived NSCs. **(C)** The pluripotent gene expression was not affected upon TrkA inhibitor treatment (2 μM, GW441756, Selleck) for 12h. Western blotting was performed in WT and Ulk1$^{-/-}$ ES lines using anti-OCT4, anti-SOX2 and anti-NANOG antibodies.
